# The effect of ketamine on eye movement characteristics during free-viewing of natural images in common marmosets

**DOI:** 10.3389/fnins.2022.1012300

**Published:** 2022-09-20

**Authors:** Zlata Polyakova, Masao Iwase, Ryota Hashimoto, Masatoshi Yoshida

**Affiliations:** ^1^Center for Human Nature, Artificial Intelligence, and Neuroscience, Hokkaido University, Sapporo, Japan; ^2^Department of Psychiatry, Osaka University Graduate School of Medicine, Suita, Japan; ^3^Department of Pathology of Mental Diseases, National Institute of Mental Health, National Center of Neurology and Psychiatry, Kodaira, Japan

**Keywords:** pharmacological model, ketamine, schizophrenia, scanpath, saccades, non-human primates

## Abstract

Various eye movement abnormalities and impairments in visual information processing have been reported in patients with schizophrenia. Therefore, dysfunction of saccadic eye movements is a potential biological marker for schizophrenia. In the present study, we used a pharmacological model of schizophrenia symptoms in marmosets and compared the eye movement characteristics of marmosets during free-viewing, using an image set identical to those used for human studies. It contains natural and complex images that were randomly presented for 8 s. As a pharmacological model of schizophrenia symptoms, a subanesthetic dose of ketamine was injected intramuscularly for transient and reversible manipulation. Eye movements were recorded and compared under a ketamine condition and a saline condition as a control. The results showed that ketamine affected eye movement characteristics during free-viewing. Saccades amplitude and scanpath length were significantly reduced in the ketamine condition. In addition, the duration of saccades was longer under the ketamine condition than under the saline condition. A similar tendency was observed for the duration of fixations. The number of saccades and fixations tended to decrease in the ketamine condition. The peak saccades velocity also decreased after ketamine injection whereas there was no difference in the main sequence relationship between saccades amplitude and peak velocity. These results suggest that ketamine affected visual exploration but did not affect the oculomotor aspect of saccades in marmosets, consistent with studies in patients with schizophrenia. Therefore, we conclude that the subanesthetic dose of ketamine is a promising pharmacological model of schizophrenia symptoms in common marmosets and can be used in combination with free-viewing paradigms to establish “translatable markers” for schizophrenia in primates.

## Introduction

Schizophrenia is known to affect one percent of the population and is classified as a mental disorder with psychosis (Andreasen, 1995). The brain mechanism of this disorder remains largely unknown and there is a need to identify behavioral markers that can be used in pathophysiology at the neuronal level using animal models. Human studies have reported dysfunction of eye movements in subjects with schizophrenia in various tasks (Holzman et al., 1973; Kojima et al., 1992; Levy et al., 1993; Arolt et al., 1998; Campana et al., 1999). Impaired visual information processing and abnormal eye movements during free-viewing of naturalistic images have been shown to be possible neurophysiological biomarkers of schizophrenia (Bestelmeyer et al., 2006; Benson et al., 2007; Miura et al., 2014; Yoshida et al., 2015). Common features of schizophrenic subjects associated with atypical visual exploration of images have been reported: prolonged fixation duration, decreased saccade amplitude, decreased saccade frequency, and decreased scanpath length (Morita et al., 2017). Increased salience-guided eye movements were reported in subjects with schizophrenia (Yoshida et al., 2015). Therefore, we hypothesize that the visuo-cognitive impairments during free-viewing reported in patients with schizophrenia may be used as an indicator in animal model of schizophrenia symptoms.

A promising non-human primate model for investigating cognitive dysfunction in neuroscientific studies is a common marmoset (*Callithrix jacchus*) model (Okano et al., 2012). The possibility of genetic manipulations and brain structure (e.g., lissencephalic cortex) of this species has multiple advantages for electrophysiological and optical imaging neuroscientific studies (Hung et al., 2015; Sadakane et al., 2015; Yamada et al., 2016; Ebina et al., 2018; Kondo et al., 2018). Marmosets, like macaques and humans, have the potential to be widely adopted for oculomotor research due to their similar saccade kinematics and their ability to perform psychophysical tasks (Mitchell et al., 2014; Chen et al., 2021). Thus, establishing an experimental paradigm for studying schizophrenia using common marmosets has multiple scientific benefits.

The motivation for the choice of free-viewing as a behavioral task is based on the fact that a significant amount of human studies on subjects with schizophrenia reported changes in eye movement characteristics during the observation of images. In addition, this type of task does not require pre-training or high cognitive capacity. Visual behavior of marmosets is comparable to that of other primates (Mitchell and Leopold, 2018) and eye movement monitoring experiments under head restraint are already well developed (Mitchell et al., 2014, 2015; Chen et al., 2021; Kaneko et al., 2022). Therefore, in the current study, we used a free-viewing task with natural and complex images presented to head-restrained marmosets. To date, there are no studies that directly compare eye movement characteristics of marmosets as a pharmacological model of schizophrenia symptoms with those of patients with schizophrenia, because of differences in visual stimuli. In the present study, we addressed this issue by choosing the same natural and complex images used in the human studies (Miura et al., 2014; Morita et al., 2017, 2019).

There are multiple approaches to establish animal models of schizophrenia (Carpenter and Koenig, 2008), which can be classified into four categories: developmental, pharmacological, lesion, or genetic manipulation (Jones et al., 2011). In the current study, we prioritized the presence of a reversible effect, which is possible in drug-induced models. Human data have shown that *N*-Methyl-D-aspartate (NMDA) antagonists such as ketamine can produce clinical symptoms of schizophrenia (Krystal et al., 1994). Pharmacological models with ketamine have been reported to produce both positive and negative symptoms of schizophrenia (Beck et al., 2020). Early human studies showed that high doses of ketamine strongly affected characteristics of eye movements (Radant et al., 1998), and it was subsequently shown that even low doses reproduce some of the abnormalities seen in subjects with schizophrenia, such as reduced eye acceleration during initiation, reduced closed loop gain (Weiler et al., 2000). Injections of subanesthetic dose of ketamine 0.3–0.5 mg/kg in non-human primates reproduce various sensory and cognitive dysfunctions of schizophrenia, such as dysfunction of the visual working memory (Sawagashira and Tanaka, 2021) and impaired visual contextual integration (Schielke and Krekelberg, 2021) in macaques, deficits in executive function in marmosets (Kotani et al., 2016). Recently, it has been reported in marmosets that ketamine also induced disruption of scan paths, with limited effects on saccade motor control during free-viewing of face images (Selvanayagam et al., 2021). However, there is no study to date examining the effect of ketamine during free-viewing of natural and/or complex images with various categories. To fill this gap, the present study examined whether subanesthetic doses of ketamine reproduce schizophrenia-like oculomotor symptoms in common marmosets, and directly compared changes in eye movement characteristics with data from human studies conducted using the same task and image dataset (Miura et al., 2014; Yoshida et al., 2015).

## Materials and methods

### Animals

The experimental protocols were approved by the Institutional Animal Care and Use Committee of the National Institutes of Natural Sciences and Hokkaido University Animal Care and Use Committee, and all experiments were performed in accordance with the guidelines of the National Institutes of Health *Guide for the Care and Use of Laboratory Animals*. Five marmosets (*Callithrix jacchus*) were subjects in this study. *Marmoset U* (8 years, 360 g), *Marmoset E* (3 years, 390 g), *Marmoset G* (5 years, 370 g), and *Marmoset J* (4 years, 410 g) were males. *Marmoset F* was a 9 years old female and weighed 460 g. When animals were not in active use, they were housed in individual cages and had *ad libitum* access to food and water. During the experiments, the animals were under the close supervision of university veterinarians and/or experimenters.

### Marmosets’ preparation

First, each marmoset was trained to sit in a marmoset chair (MMC-3505, O’Hara & Co., Tokyo, Japan) quietly for 15 min. During this first stage of chair training experimenter periodically rewarded the animal with a small piece of *Baumkuchen* cake. After animals were conditioned to be handled by the experimenter and to the movement restrictions, each marmoset underwent an aseptic surgical operation under isoflurane anesthesia. The head-holder implant (Reinforced plastic) was mounted using Estecem II (Tokuyama Dental) and used to stabilize the head during eye-tracking data collection. Then, several sessions of chair training under head-fixed conditions were repeated to give time for animals to adapt to a new restrained condition and stay calm for 30 min while licking continuously administered sweetened water. After these procedures, a free-viewing task was conducted.

### Behavioral task

#### Free-viewing stimuli

**FIGURE 1:**
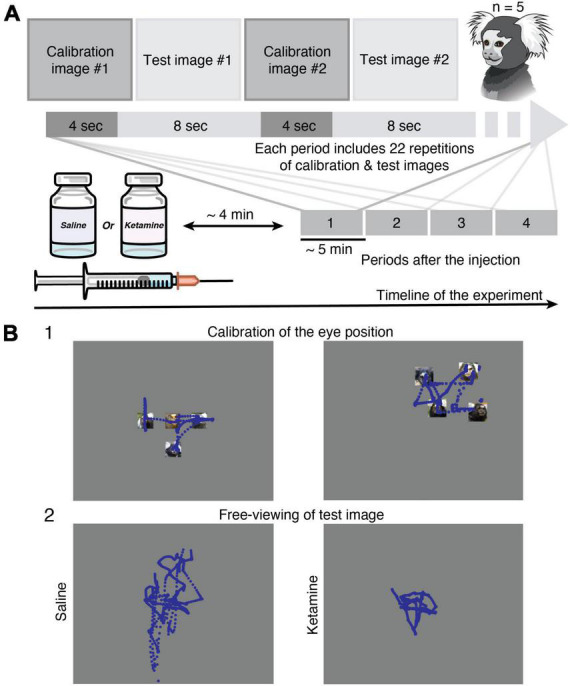
Methods. **(A)** Experimental design. Eye tracking in the free-viewing task was implemented in head-restrained marmosets (*n* = 5). Injection of saline or ketamine to marmosets was followed by a short time gap (approximately 4 min) to avoid eye nystagmus in the case of drug application. The data collection was performed in four time periods. Each period (approximately 5 min) includes a set of 22 calibration and 22 test images presented in the queue order. **(B)** Examples of gaze (blue traces) in *Marmoset G* during free-viewing of images. **(1)** Two representative examples of calibration images (see section “Materials and methods”) used for the eye position offset and gain adjustment. **(2)** Example of eye tracking data during free-viewing of the same test image from the category of faces after saline (left) and ketamine (right) injections. The test images are replaced with a gray rectangle. See section “Materials and methods” for detail.

In the present study, we used the same test pictures data set as for human studies (Yoshida et al., 2015; Morita et al., 2017), which allows us to perform a direct comparison of eye movement characteristics reported for human subjects with schizophrenia and data collected on marmoset with the pharmacological model of the disorder. The dataset of 56 natural and complex images in seven categories (nature, animal, face, building, daily, food, geometric, noise) was used in the study. Face images were selected from Matsumoto and Ekman database (Matsumoto and Ekman, 1988) and the rest from the International Affective Pictures System (IAPS) (Lang et al., 2008), which are not allowed to be published in scientific journals. Test images were randomly presented for 8 s, interleaved with calibration pictures with marmoset faces in different positions presented for 4 s (Figure 1A). The gaze calibration was performed using images with four small pictures of marmoset faces located in the corners or smaller versions of the same pictures grouped in the corner/center (Figure 1B). Task conditions were similar to the one used in the human studies (Miura et al., 2014; Morita et al., 2017) with slight minor modifications (e.g., calibration procedure) for studying non-human primates.

During data collection, marmosets sat in the marmoset chair with its head fixed using the head-holder. The LCD monitor (Dell P1917S, 375 mm × 300 mm, 60 Hz) was placed at a distance of 40cm in front of the marmosets. The resolution of the screen was 1280 × 1024 pixels. Images were presented in the full-screen mode. The marmosets continuously received drops of sweetened water in order to maintain an arousal state and attention.

#### Eye tracking

Eye movements during free-viewing were binocularly recorded at 500 Hz with the EyeLink 1000 Plus eye-tracker (SR Research, Mississauga, ON, Canada). The eye position was detected based on pupil tracking and corneal reflection information available in the Eyelink system. The blink segments detected automatically by Eyelink were recorded and used for further exclusion of the data with artifacts.

### Injections

We injected a subanesthetic dose of ketamine 0.5 mg/kg (Daiichi-Sankyo Co., Tokyo, Japan) intramuscularly for transient and reversible manipulation of schizophrenia symptoms. The dosage of ketamine was determined based on previous pharmacological studies in monkeys (Pouget et al., 2010; Sawagashira and Tanaka, 2021) and marmosets (Selvanayagam et al., 2021), so that marmosets could perform the free-viewing task. To avoid the development of the drug tolerance and cumulative effect, injections of ketamine were performed once a week. The saline injections were performed on different days in between ketamine injections and were used as a control condition for further comparison. The recording of eye movements was started after the strong effect of nystagmus was diminished and covered approximately 25 min time interval after the injection (Figure 1A).

### Data analysis

The aim was to evaluate the oculomotor behavior of marmosets during free-viewing in a pharmacological model of schizophrenia symptoms. We performed a detailed analysis of pupil changes, gaze behavior, and saccade kinematics in control and modeled schizophrenic states.

#### Pupil changes

Shortly after the ketamine injection, we observed an increase in pupil size that lasts approximately 10–15 min. Since the pupil effect did not affect the eye detection quality, data reordered during pupil dilation were included in the analysis and used as an indicator of a successful drug administration.

#### Eye movement detection

EyeLink recorded gaze position and pupil diameter, which were used for the detailed analysis of eye movement characteristics. First, preprocessing of recorded data was done using MATLAB code written in-house. It includes a selection of time intervals without blinks and strong nystagmus caused by ketamine injection. Then, data were examined using GUI as described elsewhere (Chen and Hafed, 2013). Briefly, the eye movement velocities and accelerations were calculated using a fourth-order 21-Hz differentiating Savitzky-Golay smoothing FIR (Yoshida et al., 2012). Then we identified time samples of the movement in which radial eye velocity exceeded a threshold of 15°/s to exclude the noise. The start and end of saccades were defined based on the acceleration threshold of 450°/s^2^ (Chen et al., 2021; Selvanayagam et al., 2021). In addition, the minimal duration of saccades was set 15 ms and the minimal intersaccadic interval 10 ms. Finally, we manually verified all detected saccades. In addition, intervals of noise artifacts were marked by the experimenter and removed from the data. After all procedures mentioned above, the fixations were identified using self-written code as intervals of data without artifacts, blinks, and saccades with a threshold of minimal duration 50 ms.

#### Calculation of eye movement characteristics

In the present study, we evaluated parameters of saccades kinematics in the control saline condition and modeled schizophrenic state. The number of fixations/saccades was evaluated as a number of events within test image presentation time (8 s) excluding the time of blinks and noise. Duration of fixations/saccades similarly was normalized by eight seconds time interval of active viewing. Saccades amplitude in visual degrees and peak velocity (degrees per second) were calculated for each saccade and used further for the investigation of the main sequence relationship. Scanpath length was evaluated for each test image as the summation of amplitudes of saccades detected within the image presentation. The described parameters were averaged for each test image presentation case and then the median value with median absolute deviation was calculated among all images presented for each animal under the specific condition (saline, ketamine).

#### Main sequence

To characterize the main sequence, we fitted the amplitude versus the peak velocity of the saccades collected during free-viewing with the following equation (Leigh and Zee, 2015): *V* = *V*_*max*_ × {1 – exp. (–*A*/*C*)}, where (*A*) is the amplitude and (*V*) the peak eye velocity of the saccades. Parameters of the main sequence *V*_*max*_ and *C* were optimized.

#### Statistics

All statistical analyses were performed using MATLAB (Mathworks, Natick, MA, USA). Comparisons between median values of eye movement characteristics with saline and ketamine injections were performed using the two-sample Wilcoxon rank-sum test. The effect size of the estimated differences was quantified by Cliff’s delta (Δ). The general guidelines for interpretation was the following: negligible for |Δ| < 0.147, small for 0.147 ≤ |Δ| < 0.33, medium for 0.33 < = |Δ| ≤ 0.474, and large for |Δ| ≥ = 0.474 (Romano et al., 2006). A false discovery rate (FDR) with α = 0.05 was used for the *post hoc* correction (Benjamini and Hochberg, 1995).

For a statistical test of the main sequence relationship, a bootstrap resampling method was used. To test the null hypothesis in which the *V*_*max*_ for ketamine condition and saline condition are the same, the data set was resampled from merged population and fitting was done to obtain *V*_*max*_ for the resampled ketamine population and the resampled saline population. By repeating resampling procedures 999 times, the distribution of the difference between *V*_*max*_ for the resampled ketamine population and the resampled saline population was obtained. Then p value was calculated as the probability of resampled differences higher (or lower) than that of actual difference. To make the *p* values for a two-sided test, the *p* values were multiplied by two.

Differences among parameters for all five marmosets were evaluated using two repeated measures analyses of variance (ANOVA) with factors: marmoset and injection type (saline, ketamine). To evaluate the overall effect of injection type across all animals, a model without interaction term was used. Changes related to the time period were evaluated using three-way ANOVA with marmoset, injection type, and time period factors. In the case of image category dependency, we performed a three-way ANOVA with factors: marmoset, injection type, and image category.

## Results

### Overview of recorded data

In the present study, we recorded free-viewing data during the presentation of natural and/or complex images from five common marmosets (Figure 1A). The number of recording sessions per injection condition is indicated in Table 1. Each recording session included four time periods (one period ∼ 5 min; Figure 1A), however in some of them the eyes detection was not reliable due to marmosets’ behavior or the effect of injection, thus was excluded from the analysis (Table 1). Moreover, the ketamine injection induced rapid, involuntary eye movements (nystagmus) within 2–4 min which was excluded from the data analysis. The total number of images with active viewing for five animals was 1006 in the control saline case and 1046 in the ketamine pharmacological model condition. In the analysis of eye movement characteristics described below, the values were averaged for each image. Saccades were reliably detected in all animals before and after ketamine injection. The number of saccades is summarized in Table 1.

**TABLE 1:** Database overview.

Marmoset	# of sessions		# of periods		# of images		# of saccades	
	Saline	Ketamine	Saline	Ketamine	Saline	Ketamine	Saline	Ketamine
*F*	5	4	18	14	361	213	4747	2978
*U*	4	4	8	7	171	126	2889	2068
*E*	2	3	8	11	187	233	3808	4758
*G*	2	4	8	16	200	357	4795	9168
*J*	1	2	4	8	87	117	1557	2485
Total	14	17	46	56	1006	1046	17796	21457

The number of sessions, periods, images, and saccades are indicated for each marmoset and injection condition.

### Example from one animal

**FIGURE 2:**
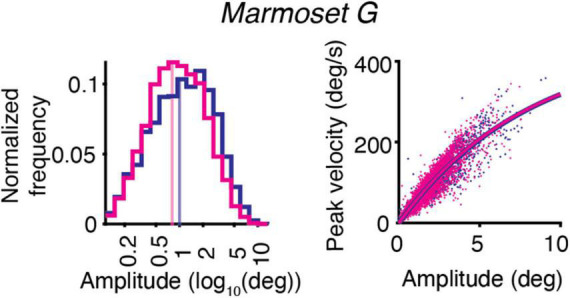
Example of the effect of ketamine (magenta) vs. saline (blue) on eye movements for *Marmoset G*. Saccade kinematics during free-viewing of natural images. **(Left)** Saccades amplitudes for two conditions plotted on a log scale. The median of the data distribution for two conditions is indicated by a shaded vertical line. **(Right)** Main sequence relationship. The saccade amplitude vs. the peak velocity is plotted separately for saline and ketamine cases. Dots indicate all saccades collected during the presentation of images. The curve represents the fitting of the data to the equation described in section “Materials and methods.”

The effect of ketamine on the distribution of saccades amplitudes is shown for one animal – *Marmoset G* (Figure 2, *left*). The example data demonstrates the decrease in saccades amplitudes after ketamine injection, in comparison to saline injections. On the other hand, the main sequence relationship, which is represented by plotting the peak velocity and the saccade amplitude of each saccade, was overall unaffected by ketamine injection (Figure 2, *right*).

### Statistical analysis of eye movement characteristics

**FIGURE 3:**
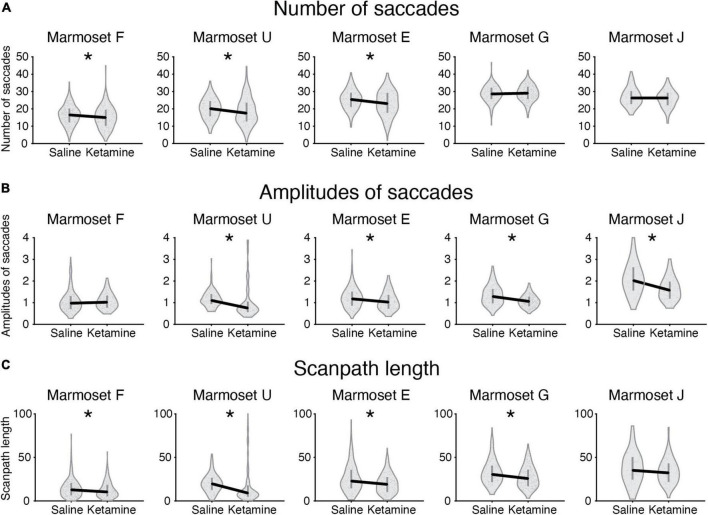
Eye movement characteristics in control saline case and ketamine pharmacological model condition plotted for five marmosets. Median values for two conditions plotted (black solid line) for the following characteristics: **(A)** Number of saccades; **(B)** Amplitudes of saccades; **(C)** Scanpath length. The values averaged per picture are indicated as a dot on a violin plot. Error bars denote the first and the third quartile. Significant cases after FDR *post hoc* correction are indicated by an asterisk.

The analysis of major eye movement characteristics was performed for the saline control case and ketamine pharmacological model condition in five marmosets (Table 2 and Figure 3).

**TABLE 2:** Eye movement characteristics in the control condition and the ketamine condition in five marmosets.

Condition		Saline		Ketamine		Statistics			
Eye movement characteristics	Marmoset	Median	MD	Median	MD	*p*-value	*z* value	Ranksum	Δ
Number of fixations	*F*	11.00	3.33	9.89	2.92	**1.77 × 10^–2^**	2.37	113078	–0.32
	*U*	11.71	2.82	12.21	4.87	0.16	–1.41	24618	–0.28
	*E*	21.78	3.60	20.99	4.50	**9.88 × 10^–3^**	2.58	43274	0.02
	*G*	22.38	3.93	22.72	3.98	0.54	–0.61	58089	–0.10
	*J*	24.36	4.06	23.12	3.14	**2.63 × 10^–2^**	2.22	10192.5	0.09

Number of saccades	*F*	16.52	3.58	14.98	4.35	**9.16 × 10^–3^**	2.61	118524.5	–0.30
	*U*	20.15	4.13	17.48	4.78	**1.22 × 10^–2^**	2.51	28557	–0.04
	*E*	25.43	3.58	23.01	5.19	**1.19 × 10^–3^**	3.24	44655	0.06
	*G*	28.58	3.09	29.05	3.18	0.38	–0.87	57586	–0.11
	*J*	26.25	3.52	26.26	3.29	0.30	1.04	9675.5	0.00

Duration of fixations	*F*	483.07	289.89	503.39	219.43	**4.58 × 10^–3^**	–2.84	87696	–0.14
	*U*	340.42	113.15	436.18	271.19	**4.49 × 10^–3^**	–2.84	22359.5	–0.19
	*E*	247.11	73.97	284.02	126.59	**2.32 × 10^–2^**	–2.27	34565	–0.13
	*G*	210.21	52.81	204.03	39.56	0.27	1.11	57255.5	0.06
	*J*	217.40	50.59	234.65	58.75	5.50 × 10^–2^	–1.92	6516	–0.16

Duration of saccades	*F*	64.70	20.35	60.79	11.07	0.67	0.42	87696	0.02
	*U*	52.11	6.13	59.44	9.03	**6.32 × 10^–14^**	–7.50	22359.5	–0.51
	*E*	66.93	8.54	72.76	13.17	**6.65 × 10^–6^**	–4.51	34565	–0.26
	*G*	59.99	7.19	61.71	5.78	**8.33 × 10^–5^**	–3.94	57255.5	–0.20
	*J*	68.77	12.95	72.79	7.67	**2.57 × 10^–5^**	–4.21	6516	–0.34

Saccades amplitude	*F*	0.98	0.28	1.02	0.24	0.17	–1.37	101151	–0.07
	*U*	1.11	0.21	0.75	0.19	**3.23 × 10^–11^**	6.64	28154	0.25
	*E*	1.18	0.30	1.03	0.28	**2.08 × 10^–3^**	3.08	43171	0.17
	*G*	1.29	0.32	1.06	0.20	**4.49 × 10^–12^**	6.92	68411	0.35
	*J*	2.02	0.55	1.57	0.36	**5.52 × 10^–7^**	5.01	11006	0.41

Scanpath length	*F*	12.80	6.50	10.34	5.29	**1.11 × 10^–2^**	2.54	118380	0.12
	*U*	19.81	6.56	9.00	4.36	**6.33 × 10^–10^**	6.18	31443	0.41
	*E*	22.89	9.51	19.08	7.89	**2.41 × 10^–4^**	3.67	45207	0.21
	*G*	30.56	8.64	25.74	8.69	**3.48 × 10^–5^**	4.14	64868.5	0.21
	*J*	35.23	12.64	32.30	9.98	0.11	1.58	9913	0.13

Peak velocity	*F*	42.85	14.00	44.23	12.37	0.23	–1.21	101471	–0.06
	*U*	55.75	9.99	33.27	9.28	**1.51 × 10^–11^**	6.75	29536	0.38
	*E*	49.29	13.42	44.25	13.17	**1.22 × 10^–2^**	2.51	42461	0.14
	*G*	63.29	13.72	52.87	9.80	**8.77 × 10^–11^**	6.49	67620	0.33
	*J*	92.40	18.90	71.58	15.29	**1.03 × 10^–5^**	4.41	10757	0.36

Median value with median absolute deviation indicated for each characteristic. Two-sample Wilcoxon rank-sum test was performed for injection conditions. The effect sizes were determined by Cliff’s delta (Δ). The *p* value in bold font indicates significance after FDR *post hoc* correction performed across eye movement characteristics and monkeys (7×5).

#### Number of fixations/saccades

We compared the number of fixations/saccades in the saline and ketamine injection conditions. The number of fixations significantly decreased in three marmosets (*Marmosets F, E, J*; Table 2). Similar tendency was observed for the number of saccades (*Marmosets F, U, E*; Table 2 and Figure 3A) in the ketamine condition. A two-way ANOVA was conducted that examined the effect of ketamine and saline injections on the number of fixations/saccades for the data from all animals. The significant difference was found in both cases of number of fixations [*F*(1,2126) = 4.05, *p* = 4.42 × 10^–2^] and number of saccades [*F*(1,2152) = 18.07, *p* = 2.22 × 10^–5^].

#### Duration of fixations/saccades

Duration of fixations had a tendency to increase after ketamine injection in four animals and was significant in three (*Marmosets F, U, E*; Table 2). Duration of saccades was increased significantly in four marmosets out of five (*Marmosets U, E, G, J*; Table 2). We conducted repeated measures ANOVA and observed significant effect of ketamine over saline for both duration of fixations [*F*(1,2002) = 13.07, *p* = 3.07 × 10^–4^] and saccades [*F*(1,2046) = 15.06, *p* = 1.07 × 10^–4^].

#### Saccades amplitude and scanpath length

The analysis of eye movements revealed that the mean saccade amplitudes were reduced in four marmosets after injection of ketamine (*Marmosets U, E, G, J*; Table 2 and Figures 2, 3B). We confirmed the presence of a significant decrease in saccade amplitudes after ketamine injection using two-way ANOVA for data from five marmosets [*F*(1,2034) = 66.35, *p* = 6.51 × 10^–16^].

Scanpath length was reduced significantly in four animals (*Marmosets F, U, E, G*; Table 2 and Figure 3C). A two-way ANOVA for all marmosets confirmed the presence of significant differences in scanpath length between ketamine and saline injection conditions [*F*(1,2139) = 36.61, *p* = 1.7 × 10^–9^].

#### Peak velocity and main sequence relationship

Peak velocity significantly decreased in four animals (*Marmosets U, E, G, J*; Table 2) in the ketamine condition. ANOVA results for the full dataset supported this finding [*F*(1,2042) = 58.84, *p* = 2.64 × 10^–14^].

The slope of the main sequence relationship between the peak velocities and the amplitudes of saccades was not altered by ketamine injection (Figure 2, right). Estimated parameters of the main sequence relationship V_*max*_ and C were similar across marmosets in the case of saline injection and not significantly different from parameters optimized for results after ketamine injection (Table 3).

**TABLE 3:** Optimized parameters of the main sequence relationship (see section “Materials and methods”) for each marmoset.

Condition		Saline		Ketamine		*p*-value
Marmoset		Estimate	*SE*	Estimate	*SE*	
*F*	V_max_	781.43	47.09	560.58	39.344	0.19
	C	16.61	1.0929	11.598	0.90856	0.19

*U*	V_max_	571.71	46.663	2175.1	296.01	0.11
	C	10.701	0.97626	48.269	7.0578	0.09

*E*	V_max_	393.3	16.195	334.57	11.349	0.26
	C	8.0851	0.3988	6.8396	0.27713	0.32

*G*	V_max_	507.19	26.41	396.15	12.864	0.96
	C	9.9307	0.63472	7.1088	0.29272	0.88

*J*	V_max_	448.43	10.884	445.44	9.8733	0.22
	C	8.1007	0.23769	7.9631	0.20702	0.16

Estimated value and standard error indicted for each injection condition. The *p*-value for a difference between saline and ketamine models parameters indicated for a two-sided test.

All eye movement characteristics analyzed in the present study significantly changed after administration of ketamine to marmosets, except for main sequence relationship parameters. The effect size (Cliff’s delta) for the majority of significant changes was within a range of small and medium guideline intervals (see section “Materials and methods”). For other cases, there were no significant differences.

### Duration of the ketamine effect

**FIGURE 4:**
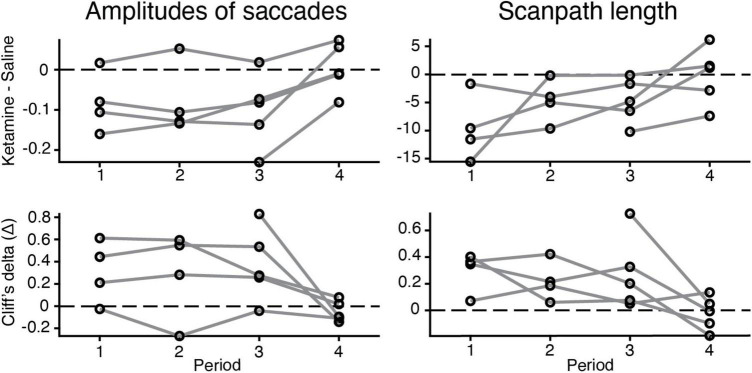
Changes in the eye movement characteristics within time periods after the injection (saline vs. ketamine). Example of alterations in amplitudes of saccades (log values) and scanpath length. **(Up)** the difference between median values of ketamine and saline cases indicated (black circle) for each time period and animal (gray line). **(Down)** Cliff’s delta was used to quantify the effect size of the estimated difference between median values in two injection conditions. For interpretation, we followed the general guidelines described in section “Materials and methods.”

Our experimental data demonstrate that the major differences in eye movement characteristics were in place within three time periods after ketamine injection and the effect was reduced in the fourth period (Figure 4). Three-way ANOVA demonstrated presence of statistical significance in the interaction between injection type and time period, for all characteristics related to saccades [duration of saccades *F*(3,2024) = 3.14, *p* = 2.46 × 10^–2^; saccades amplitudes *F*(3,2008) = 12.04, *p* = 8.18 × 10^–8^; peak velocity *F*(3,2020) = 11.03, *p* = 3.5 × 10^–7^; scanpath length *F*(3,2117) = 12.3, *p* = 5.59 × 10^–8^], except for the number of saccades. This finding indicates that the effect of ketamine is altered across time periods. The example data for saccades amplitudes and scanpath length in five animals illustrates that the effect of ketamine was reduced in the fourth period (Figure 4).

### Effect of image category

**FIGURE 5:**
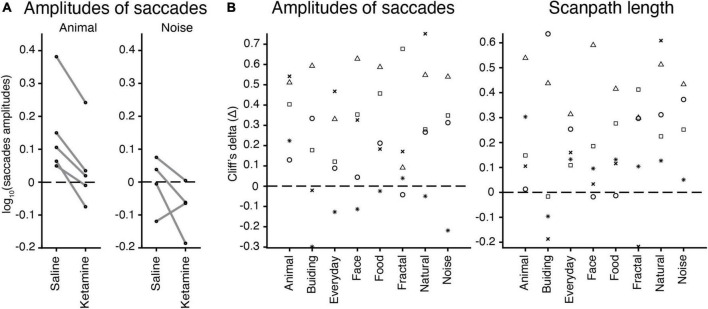
The effect of image categories on the eye movement characteristics. **(A)** Example of saccades amplitudes after saline or ketamine injection for ‘animal’ and ‘noise’ image categories. Median values for a log of saccades amplitudes are indicated (black circle) and connected in two conditions for each animal (gray line). Note: *Monkey J* has not been presented with ‘noise’ images. **(B)** Effect sizes (Cliff’s delta, see section “Materials and methods”) for the test of difference in amplitudes of saccades (log values) and scanpath length median values in each image category are indicated by a symbol for each marmoset: *F*, asterisk; *U*, triangle; *E*, circle; *G*, square; *J*, cross.

We checked whether there is a relation between an image category (nature, animal, face, building, daily, food, geometric, noise) and eye movement characteristics in the free-viewing task. The example of saccades amplitudes in saline and ketamine conditions demonstrates higher median values for the animal image category compared to noise, as well as a decrease in saccades amplitudes after drug application (Figure 5A). Cliff’s delta (the effect size) for each animal and image category was calculated for each characteristic. There was no simple relationship found for all eye movement characteristics. The example for amplitudes of saccades and scanpath length illustrates this finding (Figure 5B). Three-way ANOVA with the interaction between injection type and image category revealed no statistical significance in eye movement characteristics. This finding suggests that the effect of ketamine is general and does not depend on specific image categories. Thus, the effect of ketamine in our study is general enough and does not depend on the choice of images. In the model without interaction, image category is statistically significant in all cases related to saccades [number of saccades *F*(7,2131) = 2.27, *p* = 2.67 × 10^–2^; saccades amplitudes *F*(7,2023) = 5.14, *p* = 8.14 × 10^–6^; peak velocity *F*(7,2031) = 10.11, *p* = 1.74 × 10^–12^; scanpath length *F*(7,2132) = 7.12, *p* = 1.98 × 10^–8^], except for saccades duration. The major eye movement characteristics are dependent on image category.

## Discussion

Our main purpose was to directly compare eye movement characteristics in patients with schizophrenia and the marmoset pharmacological model of schizophrenia symptoms. The analysis of eye movement characteristics after a subanesthetic dose of ketamine (intramuscular injection) applied in marmosets revealed similar tendencies of decreased number of fixations and saccades, reduced saccade amplitude, peak velocity, and scanpath length, and increased fixation duration. In addition, the parameters of the main sequence relationship (saccade amplitude vs. saccade peak velocity) were not affected by ketamine application. These results suggest that ketamine affected visual exploration including some attentional processes, but not oculomotor activities, as argued below. These results are consistent with data reported for subjects with schizophrenia in eye tracking studies using the same image dataset (Miura et al., 2014; Morita et al., 2017, 2019) and provide evidence for the validity of the ketamine pharmacological model. The difference was observed only for saccade duration, which was increased in marmosets but decreased in humans. Overall, our results support our hypothesis that the combination of the ketamine pharmacological model in marmosets and the free-viewing task is useful to study schizophrenia.

### Visual exploration vs. oculomotor function

After the ketamine administration, there was no significant difference in the main sequence relationship between saccades amplitude and peak velocity whereas there was a disturbance in scanpath length. Whereas eye movement characteristics may reflect a variety of functions such as covert attention and target selection (Schiller and Tehovnik, 2005), the main sequence relationship has been used to assess the oculomotor dysfunctions (Gibaldi and Sabatini, 2021). These previous findings lead us to the conclusion that visual exploration possibly predominates over oculomotor functions in the ketamine model of schizophrenia symptoms. Further studies examining visual salience may help to clarify how visual attention is affected in schizophrenia, as described below.

### Free-viewing of naturalistic stimuli

In the present study, natural and complex images were used to test the pharmacological model of schizophrenia symptoms in marmosets. The dataset was identical to that used for the human studies (Miura et al., 2014; Morita et al., 2017, 2019). Naturalistic stimuli are beneficial for human and animal studies because they mimic “real life” visual inputs in the experiment. This approach retains higher ecological validity, in contrast to analogs using tightly controlled stimuli (e.g., moving dots, oriented lines, etc.). In addition, free-viewing of natural images is suitable for extending research toward the analysis of visual salience and processes behind this phenomenon because various visual features can be examined with naturalistic images. Thus, the combination of salience analysis and neurophysiology can be beneficial in studying visual attention and its pathophysiological changes (Yoshida et al., 2012).

### Ketamine as a model of schizophrenia symptoms

In the present study, subanesthetic doses of ketamine were applied as a model of schizophrenia symptoms in marmosets. Ketamine was chosen because its effect is reversible, whereas it is irreversible in other drugs such as phencyclidine (Jentsch and Roth, 1999) or methamphetamine (Featherstone et al., 2007). This was confirmed in the present study, with the effect of ketamine decreasing in the fourth period, corresponding to 20–25 min after injection (Figure 4). The three-way ANOVA also revealed a significant interaction between drug effect and period, suggesting that the effect of ketamine varied with periods. Because the effects of ketamine are reversible, it is possible to track changes in neuronal activity before and after ketamine injection, which has a potential advantage for future neurophysiological applications.

Ketamine is also known to have antidepressant effects which last up to 7 days (Zarate et al., 2006). Since the effect of ketamine on eye movement characteristics in the present study was transient and diminished in 25 min, it is unlikely that the present findings reflect the antidepressant effects of ketamine.

### The effect of ketamine on image category

As shown in Figure 5 and in the accompanying analysis, the main effect of image category was statistically significant in all cases, i.e., the eye movement characteristics depend on categories. However, the interaction between drug and image category was not statistically significant, indicating that the effect of ketamine was not affected by image category. This suggests that the effect of ketamine is robust and independent of specific image types. This finding provides flexibility in the selection of image databases for future experiments and could be important based on the literature on gaze metrics in the schizophrenia research (Hashimoto, 2021). We also note that the effect of ketamine can depend on visual salience of images and further experiments are necessary to clarify this possibility.

### Future directions

An extension of this study is to examine visual salience at gazes using the Itti-Koch saliency computational model (Itti et al., 1998; Yoshida et al., 2012). This approach may provide a better understanding of the abnormalities in attentional processes found in schizophrenic subjects and marmoset ketamine models may replicate the effect on visual salience reported in human studies (Yoshida et al., 2015). In addition to static images, movie stimulus can be adopted (Chen et al., 2021). Because movie stimuli have been reported to act simultaneously and reliably on multiple cortical processing systems (Bartels and Zeki, 2004), this approach is beneficial for neurophysiological studies of visual salience. Electrophysiological and Ca-imaging techniques that are already established for marmosets (Ebina et al., 2018; Kondo et al., 2018; Ghahremani et al., 2019; Selvanayagam et al., 2019), in combination with our experimental protocol, can be used to study neuronal activities at the cell level in pharmacological models of schizophrenia. The application of multiple approaches and the creation of a database for researchers and medical doctors can facilitate the development of both neuroscience and psychiatry (Onitsuka et al., 2022b).

### Clinical significance

Direct comparison of eye movement characteristics in marmoset and human studies conducted on the identical image data set strengthened the validity of the pharmacological model of schizophrenia. The experimental protocol developed here can be used in various neuroscientific studies on animals to provide fundamental insight into pathophysiology. Based on the findings from animal studies, new, effective eye tracking tests could be created for the early diagnosis of psychiatric disorders, including schizophrenia (Onitsuka et al., 2022a). In addition, research with non-human primates can suggest effective pharmacological manipulation strategies and targets, which can facilitate drug development and consequently contribute to psychiatry.

## Conclusion

In conclusion, our findings demonstrate that the subanesthetic administration of ketamine is a promising pharmacological model of schizophrenia in common marmosets and can be used in combination with free-viewing paradigms to establish “translatable markers” (Okano et al., 2016) of schizophrenia in non-human primates. Our experimental protocol can be further used for electrophysiological or optical imaging studies in simulated schizophrenic states and can provide valuable insights into the involvement of certain brain regions in cognitive functions and visual information processing. In addition, details of changes in neuronal activity in pathophysiological conditions could lead to a better understanding of the underlying processes behind neuropsychiatric disorders.

## Data availability statement

The original contributions presented in this study are included in the article/supplementary material, further inquiries can be directed to the corresponding author.

## Ethics statement

The animal study was reviewed and approved by Hokkaido University Animal Care and Use Committee.

## Author contributions

MY designed the research. MY and ZP performed the research and analyzed the data and wrote the manuscript. MI and RH provided the images. All authors contributed to the article and approved the submitted version.
